# Trichostatin A inhibits radiation-induced epithelial-to-mesenchymal transition in the alveolar epithelial cells

**DOI:** 10.18632/oncotarget.21664

**Published:** 2017-10-09

**Authors:** Devipriya Nagarajan, Lei Wang, Weiling Zhao, Xiaochen Han

**Affiliations:** ^1^ Department of Radiation Oncology, Wake Forest School of Medicine, Winston-Salem, NC, USA; ^2^ School of Chemical & Biotechnology, SASTRA University, Thanjavur, Tamil Nadu, India; ^3^ Tangshan People's Hospital, Tangshan, Hebei, China

**Keywords:** TSA, EMT, TGF-beta, radiation, HDAC

## Abstract

Radiation-induced pneumonitis and fibrosis are major complications following thoracic radiotherapy. Epithelial-to-mesenchymal transition (EMT) plays an important role in tissue injury leading to organ fibrosis, including lung. Our previous studies have reported that radiation can induce EMT in the type II alveolar epithelial cells in both *in vitro* and *in vivo*. HDAC inhibitors are a new family of anti-cancer agents currently being used in several clinical trials. In addition to their intrinsic anti-tumor properties, HDAC inhibition is also important in other human diseases, including fibrosis and radiation-induced damage. In this study, we evaluated the effect of Trichostatin A (TSA), a HDAC inhibitor, on radiation-induced EMT in type II alveolar epithelial cells (RLE-6TN). Pre-treatment of RLE-6TN cells with TSA inhibited radiation-induced EMT-like morphological alterations including elevated protein level of α-SMA and Snail, reduction of E-cadherin expression, enhanced phosphorylation of GSK3β and ERK1/2, increased generation of ROS. Radiation enhanced the protein level of TGF-β1, which was blocked by N-acetylcysteine, an antioxidant. Treating cells with SB-431542, TGF-β1 type I receptor inhibitor, diminished radiation-induced alterations in the protein levels of *p*-GSK-3β, Snail-1 and α-SMA, suggesting a regulatory role of TGF-β1 in EMT. Pre-incubation of cells with TSA showed significant decrease in the level of TGF-β1 compared to radiation control. Collectively, these results demonstrate that i] radiation-induced EMT in RLE-6TN cells is mediated by ROS/MEK/ERK and ROS/TGF-β1 signaling pathways and ii] the inhibitory role of TSA in radiation-induced EMT appears to be due, at least in part, to its action of blocking ROS and TGF-β1 signaling.

## INTRODUCTION

Thoracic radiotherapy, either using X-rays or similar rays, is one of the successful treatment modes for lung and breast cancers. In lung cancer cases, 60% of patients receive radiation treatment either alone or in combination with surgery/chemotherapy [1]. The lung complications, including pneumonitis and fibrosis develop within month to years after treatment with radiotherapy thereby affects survival of cancer patients [2]. Myofibroblasts plays important role in fibrosis and upon activation, cells synthesize and stimulates deposition of extracellular matrix (ECM) proteins; however, recent studies focuses on epithelial cells which have ability to convert themselves into myofibroblast by a process called “epithelial-mesenchymal transition (EMT)” and it has been reported in idiopathic pulmonary fibrosis and experimental fibrosis [3, 4]. During EMT, epithelial cell loses its polarity gradually and acquires mesenchymal features with enhanced migratory potential [5].

α-SMA, a mesenchymal protein, is released in response to myofibroblast activation and confers strong contractile properties [6]. E-cadhein is an epithelial marker involved mainly in maintenance of cell-cell interaction and preserves structural integrity of the cell [7]. Snail, a master regulator of E-cadherin, helps epithelial cells to develop into fibroblast-like migratory mesenchymal cells [8].

Early studies indicate that TGF-β can partially mediate EMT through a network of signaling and transcriptional events [9, 10]. In addition to TGF-beta signaling pathways other molecules like Rho, Ras, ERK, MAPK, Wnts, NFkappaB and PI3K has been reported to enhance EMT [11]. Our *in vivo* studies also suggested that FVB/N mice upon exposure to thoracic radiation (single and fractionated) are highly sensitive to radiation and EMT might play a inter-mediatory role towards development of lung fibrosis [12, 13]. Previously, we reported that radiation stimulated EMT via ERK/GSK3s/Snail pathway in RLE-6TN cells (alveolar type II epithelial cells) [13].

Trichostatin A (TSA) is an effective inhibitor of Histone deacetylases (both class I and class II) and is isolated from Sterptomyces sp. as a fermentation product [14, 15]. TSA effectively inhibits cell cycle arrest and has been used in clinical trial for the treatment of cancer [16, 17]. On the other hand, it has been considered as a effective agent against fibrogenic diseases including liver fibrosis and cutaneous radiation syndrome [18–20]. TSA has been reported to decrease EMT induced by TGF β2 and thereby prevents the migratory potential of lens epithelial cells [21]. However, the role of TSA on gamma radiation induced alveolar EMT is not clearly investigated. In the present study, we tried to i] understand the role of TSA on gamma radiation-induced EMT in alveolar epithelial cells (RLE6TN and A549); ii] analyse cell signaling events involved in inhibitory effect of TSA on radiation-induced EMT.

## RESULTS

### TSA reversed EMT in irradiated rat alveolar epithelial cells (RLE-6TN cells)

**Figure 1:**
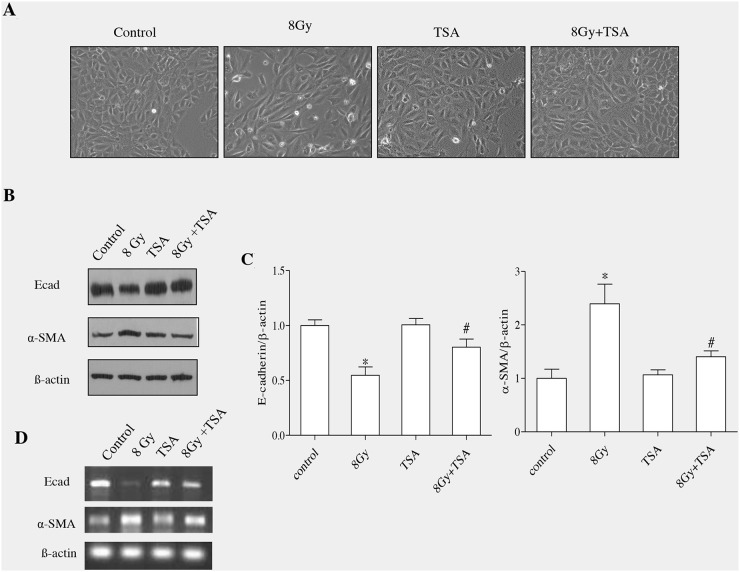
TSA inhibits EMT induced by irradiation in RLE-6TN cells **(A)** RLE-6TN cells were grown to 60% confluency in tissue culture plates and treated with TSA (100nM) for 2 h followed by radiation treatment at the dose of 8Gy. Images were captured at the magnification of 200X using inverted microscope and representative morphological changes are shown. **(B)** The protein levels of E-cadherin and α-SMA were determined using western blot analysis at 72 h post-treatment with radiation and/or. **(C)** Densitometric analysis of the Western blot results from B. Data are mean ± SEM; n = 3; ^*^ p < 0.05 vs. non-irradiated control; ^#^ p < 0.05 vs. irradiated control. The same amounts of total protein are loaded in each lane. **(D)** RT-PCR analysis of E-cadherin mRNA and α-SMA in the cells collected at 72h postirradiation.

The morphological changes induced by radiation on RLE-6TN cells have been reported previously from our lab. We observed that radiation promoted loss of cell-cell contacts in RLE-6TN and converted their structures from cuboidal to a spindle shaped fibroblastic phenotype [13]. To know the effect of TSA on morphological changes induced by radiation, we treated RLE-6TN cells with 100nM TSA prior to irradiation (single dose; 8Gy) and recorded morphologic changes of alveolar cells after 72h. Untreated RLE-6TN cells showed a cobblestone epithelial morphology and cell-cell contacts were clearly observed. But irradiated cells displayed lose of cell-cell contacts and showed more elongated spindle shaped morphology. However, pre-treatment with TSA effectively protected the epithelial cells from radiation-induced morphological changes. TSA alone treated cells did not alter the epithelial architecture of RLE-6TN cells (Figure 1A). At molecular level, EMT enhanced the expression of mesenchymal proteins (Snail, alphaSMA) and reduced expression of epithelial proteins (E-cadherin) [7].

We next investigated the role of TSA on the level of E-cadherin and α-SMA in RLE6TN cells, which were harvested at 72h post irradiation. As E-cadherin is an epithelial marker and α-SMA is a mesenchymal protein [13], in our study we evaluated both proteins using western blot analyses. Radiation (8Gy) markedly reduced the protein and gene expression of epithelial marker and also enhanced mesenchymal marker in alveolar epithelia cells (Figure 1B and 1D). However, TSA led to a significant modification in the protein and gene expressions of both E-cadherin and α-SMA in cells collected at 72 h after irradation (Figure 1B and 1D). Densitometric analysis of the Western blotting results obtained from three independent experiments showed that TSA treatment before irradiation enhanced enhanced the level of E-cadherin up to 85% and reduced α-SMA level to near normal (Figure 1C). TSA treatment alone did not induce any changes in the protein and gene expression of E-cadherin and α-SMA when compared to those from the untreated cells. Thus, morphological observations together with alteration in the epithelial and mesenchymal markers suggested that TSA treatment effectively inhibited the epithelial cells to undergo transition into mesenchymal phenotype when exposed to radiation at dose of 8Gy.

### TSA inhibited the activation of snail and phosphorylation of GSK3β

**Figure 2:**
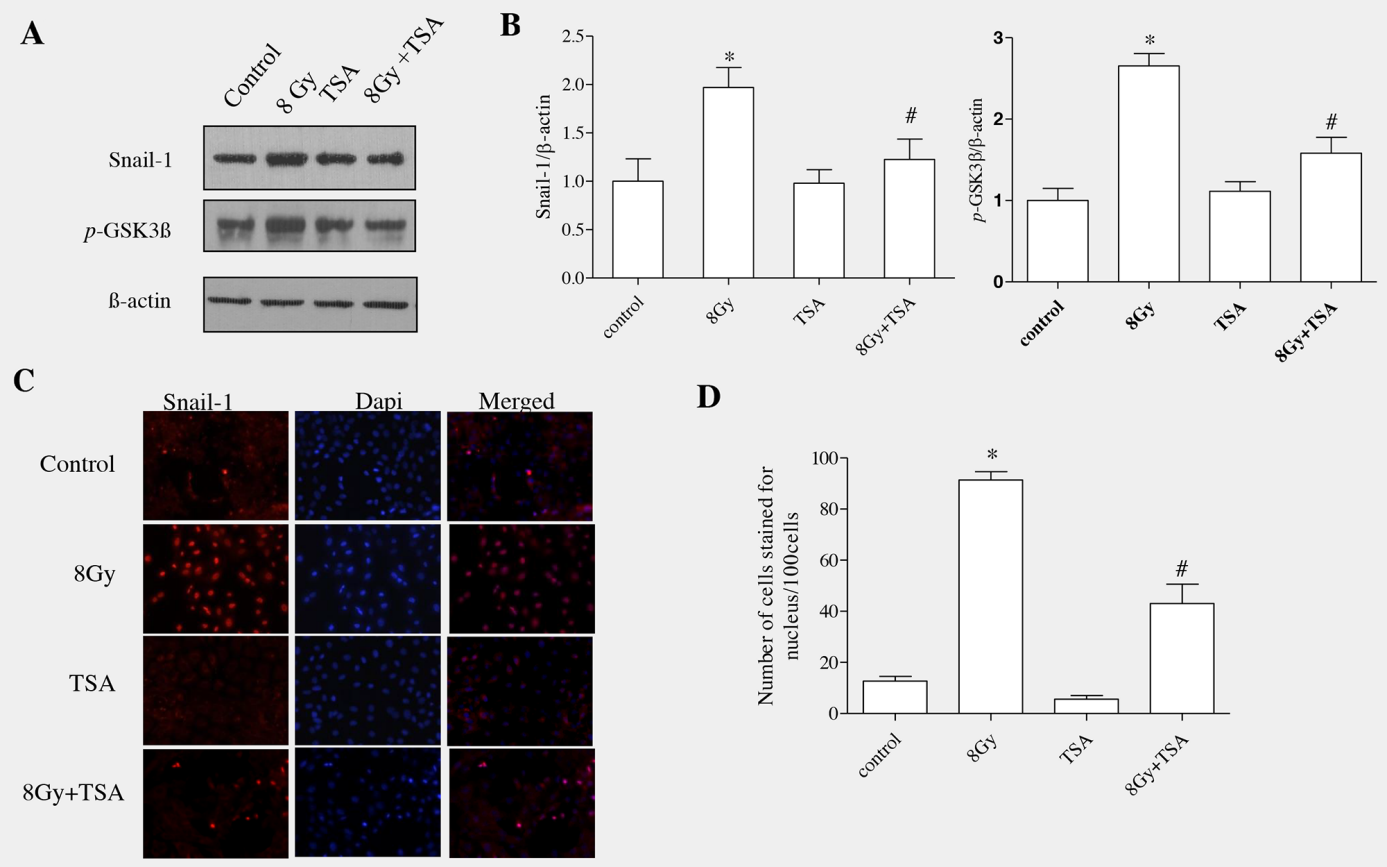
TSA reduced Snail activation and inhibited the phosphorylation of GSK3β in irradiated RLE-6TN cells RLE-6TN cells were irradiated with 8 Gy of ^137^Csγ rays, protein samples were collected at 7h post-irradiation and analysed using western blot for Snail and GSK3β(pSer9). **(A)** Shows the representative blot of Snail and GSK3β(pSer9) in RLE-6TN cells. **(B)** Shows densitometric quantification of Snail and GSK3β (pSer9); data are mean ± SEM; n = 3; ^*^ p < 0.05 vs. non-irradiated control; ^#^ p < 0.05 vs. irradiated control. **(C)** RLE-6TN cells was examined by immunofluorescence for staining of Snail (red) and Dapi (blue) in untreated and treated cells at 7 h postirradiation (400 X). Merged images of Snail and DAPI showed that radiation enhanced the nuclear colocalization of Snail where as TSA-pretreatment decreased the nuclear localization of Snail in comparison with radiation treatment. **(D)** Positively stained cells were identified using fluorescence microscope in 10 high-power fields. Quantification was determined by counting five different fields per assay. The number of cells stained for nucleus was calculated as the average of five different fields in each sample. The data were statistically analyzed from three independent experiments using one-way ANOVA.

Snail binds specifically to the promoter region of E-cadherin gene and represses its transcription; thereby snail act as an important transcriptional regulator of EMT. We next examined the effect of TSA on Snail protein in alveolar epithelial cells. RLE-6TN cells were incubated with TSA (100nM) for 2h and cells were exposed to radiation (8Gy). The cells were then harvested at 7h to determine the level of snail protein in all the groups. As shown in the Figure 2A, Snail was significantly increased in irradiated group which was effectively modulated by the treatment of TSA, as we observed from the data of western blot (Figure 2A). The mean fold change was analysed using densitometric analysis and it showed that there was decrease in the Snail protein level in control, TSA and also TSA+radiation groups (1.0±0.23, 0.98±0.14 and 1.22±0.21, respectively when compared with irradiated cells where the levels was 1.97±0.21 (Figure 2B).

Normally in cells, accumulation of Snail is controlled mainly by GSK3β (glycogen synthase kinase3 β) which tightly binds and phosphorylates Snail protein. Upon phosphorylation, Snail undergoes ubiquitination which ultimately leads to proteasomal degradation [23]. GSK-3β activity has been known to be necessary for maintaining the epithelial architecture. GSK3β activity is upregulated or downregulated depending on the phosphorylation site of aminoacid. It is upregulated when phosphorylation ocurrs at tyrosine-216; it is downregulated when phosphorylation occurs at N-terminal of serine-9 residue [24]. In our previous study, Western blot analysis on total GSK-3β, GSK-3β (pTyr216) and GSK-3β (pSer9 showed that radiation stimulated the inactive form of GSK3β(pser9), whereas the total GSK-3β and active form GSK-3β (pTyr216) remained unchanged [13]. Our current data showed that there was increased expression of pGSK3β(ser9) in irradiated group which was effectively modulated upon TSA pre-treatment (Figure 2A and 2B).

GSK3β is well known to regulate Snail expression and activation by mainly inhibiting its i] transcription; ii] stimulating its degradation and iii] preventing nuclear translocation [25]. Therefore, inhibition of GSK-3β function results in an increased expression of Snail and its translocates into nucleus where it becomes functionally active to stimulate EMT markers.

RLE-6TN cells were seeded onto 8-well chamber slides and immunofluorescence analysis was performed to know the effect of TSA on nuclear translocation of Snail. Our results showed that irradiation enhanced the nuclear translocation of Snail at 7h, whereas pretreatment with TSA reduced the radiation-induced changes (Figure 2C and 2D). Altogether, our data showed that TSA prevented GSK-3β inactivation and Snail accumulation/nuclear translocation in the irradiated cells, indicating that TSA played an inhibiting role in the key transcription factor of EMT induced by irradiation.

### TSA inhibited radiation-induced ERK activation and ROS formation in alveolar epithelial cells

The activation of GSK3β can be controlled by various mechanisms, however, phosphorylation of GSK3β via protein kinases play a crucial role in regulation. In our earlier study, our data suggested that ERK actively involved in inhibiting GSK-3β by phosphorylating at ser-9 residue during radiation-induced EMT in alveolar epithelial cells [13]. We analyzed the effect of TSA on ERK activation at 15 min of post irradiation in alveolar epithelial cells.

**Figure 3:**
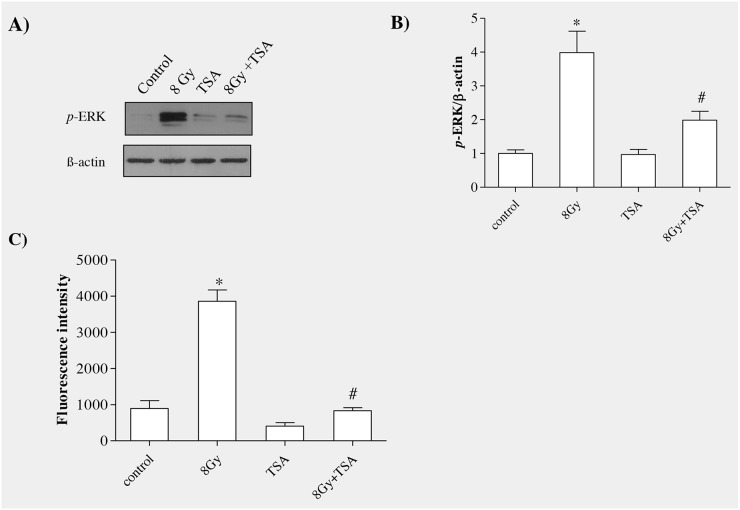
TSA inhibited radiation-induced ERK activation and ROS production in RLE-6TN cells **(A)** Western blot analysis of phosphorylation of p44/42ERK at 15mins following treatment with irradiation and/or 100 nM TSA. TSA pretreatment abolished the radiation-induced increase in the level of *p*-ERK in RLE-6TN cells. **(B)** Shows the densitometric quantification of p44/42ERK; data are mean ± SEM; n = 3; ^*^ *p* < 0.05 vs. non-irradiated control; ^#^ p < 0.05 vs. irradiated control. **(C)** ROS generation in RLE-6TN cells were determined using DCFH-DA assay, expressed as fluorescence intensity. TSA prevented radiation-induced ROS generation in RLE-6TN cells. Results are means S.E.M for at least three separate experiments. ^*^ *p* < 0.05 vs. non-irradiated control; ^#^ p < 0.05 vs. irradiated group.

Western blot analysis and and densitometric quantification showed that preincubation of cells with TSA for 2h reduced phosphorylated ERK protein levels when compared to irradiated group (Figure 3A and 3B). ROS act as a important signalling mediator during radiation induced EMT by activating ERK/GSK3β/Snail pathway in rat alveolar epithelial cells, as we reported previously [13], thus we aimed to understand whether TSA can modulate radiation-induced oxidative stress in alveolar epithelial cells. For that, 100nM of TSA was incubated 2h before irradiation (8Gy) in rat alveolar epithelial cells and ROS formation was measured using DCFH-DA assay [26]. As shown in Figure 3C, TSA effectively decreased the ROS formation when compared to the radiation control. (Figure 3C). These data indicate that TSA may act as an antioxidant to inhibit ROS production induced by radiation.

### Irradiation did not alter the levels of HDAC proteins in the RLE-6TN cells

**Figure 4:**
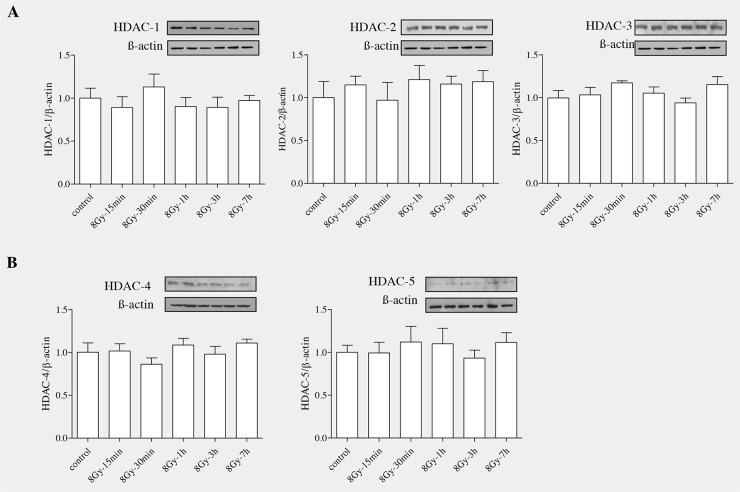
Radiation unaltered the levels of HDAC proteins in RLE-6TN cells RLE-6TN cells were irradiated with 8 Gy of ^137^Cs γ rays and protein samples were collected at various time points after irradiation for HDACs (1-6). **(A)** Shows representative blot of HDACs (1-6) levels determined using Western blot. **(B)** Shows densitometric quantification of HDACs; data are mean ± SEM; n = 3; ^*^ *p* < 0.05 vs. non-irradiated control.

Histone deactylases (HDACs) are enzymes that remove acetyl groups from lysine aminoacid in histone and also in many non histone proteins [27–29]. Based on homology, HDACs can be categorized into four different classes: Class I: HDAC1,2,3 and 8; Class II: 4,5,6,7,9 and10; Class III: Sirtunins in mammals and Sir2 in yeast; Class IV: HDAC11 [30, 31], sirtuins in mammals and Sir2 in yeast belong to class III and HDAC11 belong to class IV. As, HDACs have been shown to be involved in fibrogenesis in various organs, we expected that radiation would modulate HDAC levels in rat alveolar epithelial cells. Epithelial cells (RLE6TN) were exposed to radiation (8Gy) and were harvested at different time points (15’,30’, 1h, 3h and 7h). Western blot analysis (Figure 4A and 4B) showed that radiation didnot alter the protein levels of HDACs in irradiated RLE6TN cells in comparison with non-irradiated controls. These data indicate that radiation doesn't alter the expression of HDACs in these lung epithelial cells.

### TSA inhibited radiation-induced increase in TGF-β and CTGF proteins in RLE-6TN cells

**Figure 5:**
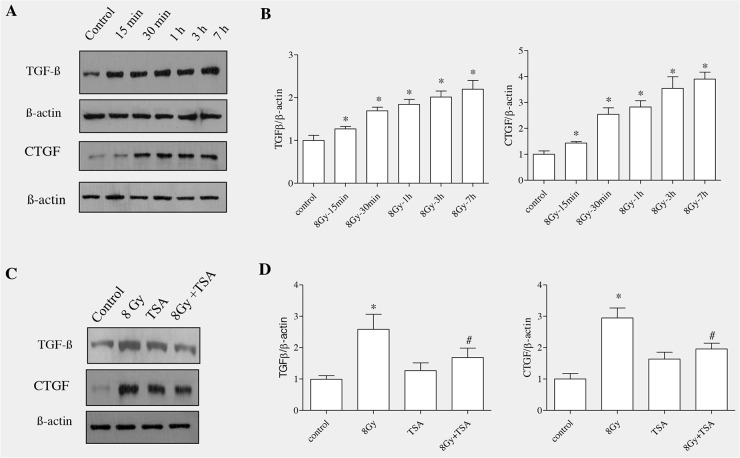
TSA inhibited radiation-induced increase in the protein levels of TGF-β and CTGF in RLE-6TN cells RLE-6TN cells were irradiated with 8 Gy of ^137^Cs γ rays and protein samples were collected at various time points after irradiation. **(A)** Shows representative blot of TGFβ1 and CTGF levels determined using Western blot. **(B)** Shows densitometric quantification of TGFβ1 and CTGF; data are mean ± SEM; n = 3; ^*^ *p* < 0.05 vs. non-irradiated control. (C) RLE-6TN cells were incubated with/without TSA for 2 h prior to irradiation with 8 Gy of ^137^Cs γ rays. Cell lysates were collected and protein levels of TGFβ1 and CTGF at 7 h post-irradiation were measured using Western blot **(C and D)**. Results are means S.E.M for at least three separate experiments. ^*^ *p* < 0.05 vs. non-irradiated control; ^#^ p < 0.05 vs. irradiated group.

TGF-β1 has been well reported to stimulate EMT in various cells including mammary epithelial cells, renal proximal tubular, lens and alveolar epithelial cells [32–35]. To determine whether TGFβ1 acts as a mediator in radiation -induced EMT in alveolar epithelial cells, TGFβ1 protein levels were collected and estimated at different time points (15min to 7h) in irradiated RLE6TN cells using Western blot analysis. The results showed that the levels of TGFβ1 were gradually increased with increase in time (Figure 5A). The mean fold increase in TGFβ1 protein at 15’, 30’, 1h, 3h and 7h post irradiation was 1.78 ± 0.28, 2.16 ± 0.19, 2.19± 0.28, 2.51 ± 0.33, and 4.03 ± 0.48, respectively, compared with that observed in non-irradiated cells 0.71 ± 0.06 (Figure 5B). Connective tissue growth factor (CTGF) involves cell migration, proliferation, adhesion and matrix production and it functions mainly as a downstream mediator of TGFβ1 signaling pathway [36]. As there was marked elevation of TGFβ1, we next evaluated the level of CTGF in alveolar epithelial cells upon irradiation. Western blot analysis and densitometric quantification of CTGF, in support of other studies, indicated that the protein levels of CTGF were increased gradually similar to the TGFβ1 in irradiated cells (Figure 5A and 5B).

In order to test whether TSA had impact on radiation-induced upregulation of TGF-β1 and CTGF, alveolar epithelial cells were incubated with TSA for two hour and exposed to radiation The results showed that TSA partially decreased the levels of TGFβ1 and CTGF in irradiated alveolar epithelial cells (Figure 5C and 5D). TSA alone did not alter the TGF-β1 and CTGF protein levels in alveolar epithelial cells.

### Incubation of SB431542, a TGFβ inhibitor, prevented EMT in irradiated epithelial cells

**Figure 6:**
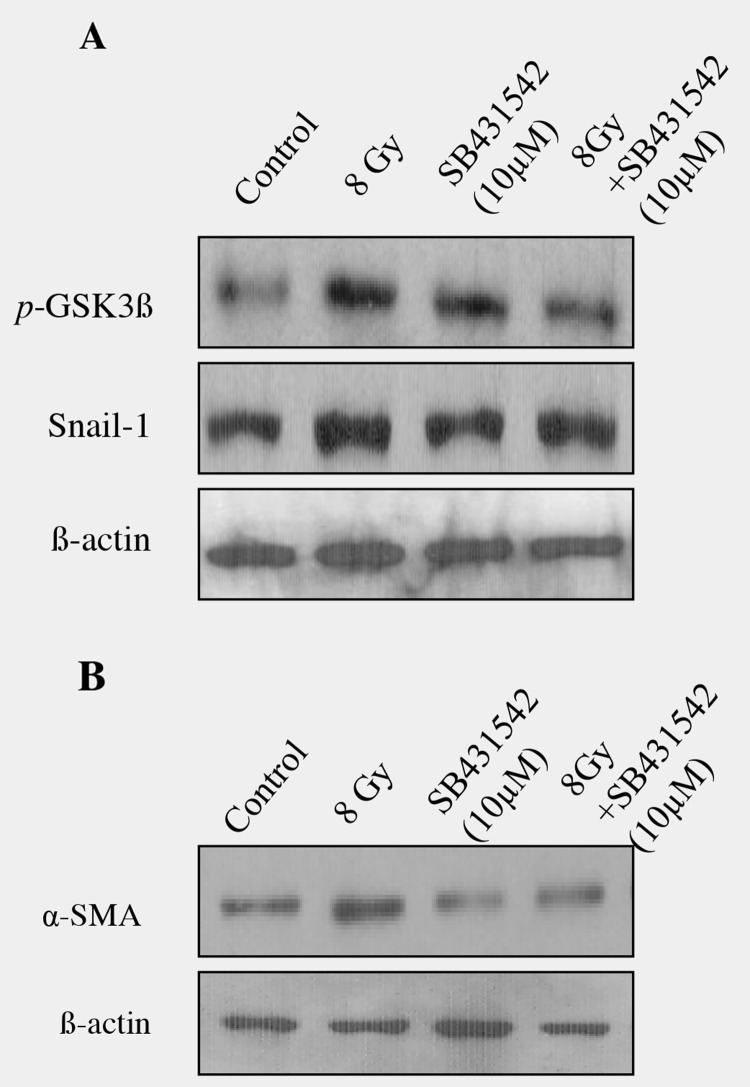
Blocking of TGF-β signaling with SB-431542 decreased radiation -induced EMT in RLE-6TNcells **(A)** RLE-6TN cells were treated SB-431542 (SB) for 2 hour prior to radiation. Cell lysates collected at different time intervals were analyzed by Western blot analysis with anti-GSK (pSer9), Snail and α-SMA antibody. An equal amount of protein loading was measured with β-actin. **(B)** Densitometric quantification showed that blocking of TGF-β signaling with SB-431542 partially reduced radiation-induced EMT in RLE-6TN cells and data are representative of 3 independent experiments. Data are mean ± SEM; n = 3; ^*^ *p* < 0.05 *vs.* non-irradiated control; ^#^ *p* < 0.05 *vs.* irradiated group.

To know if TGFβ1 plays a crucial role in radiation-induced alveolar EMT, TGF inhibitor SB431542 (10μM) was incubated for 2 hours in RLE6TN cells and exposed to radiation (8Gy) and EMT related proteins were analyzed. Western blot analysis on pGSK-3β (Ser9), Snail and α-SMA proteins showed that addition of the TGF-β1 receptor inhibitor, SB431542 significantly blocked the radiation-induced GSK-3β phosphorylation at serine 9; whereas there was a partial decrease in the Snail protein in irradiated rat alveolar epithelial cells (Figure 6A). Moreover, inhibition of TGF-β by SB-431542 also reduced the radiation-induced increase of α-SMA (Figure 6B). Our results demonstrate that inhibition of TGFβ1 effectively prevented the transition of epithelial cell to mesenchymal cells and it showed that TGF signalling, at least in part, is necessary for radiation-induced EMT.

### ROS scavenging decreased radiation-induced upregulation of TGFβ 1 in alveolar epithelial cells

**Figure 7:**
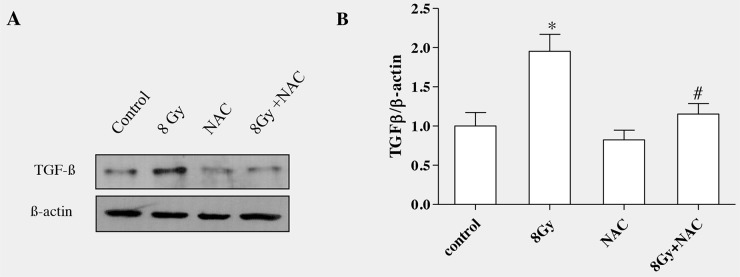
ROS scavenging decreased radiation-induced TGF-β1 level in RLE-6TN cells RLE-6TN cells were incubated with/without NAC (5mM) for 4h prior to irradiation with 8 Gy. The protein level of TGF-β1 was determined in the cell lysate at 7h postirradiation. **(A)** Shows representative blot of TGF-β1. Decreasing ROS production reduced the protein level of TGF-β1 in irradiated RLE-6TN cells. **(B)** Shows the densitometric quantification of three independent samples. The results are mean ± SEM; n = 3; ^*^ *p* < 0.05 *vs.* non-irradiated control; ^#^ *p* < 0.05 *vs.* irradiated group.

Studies have shown that ROS converts the latent form of TGFβ in to active form, therefore inhibition or neutralization of ROS can decrease TGFβ activity [37]. We have reported radiation-induced generation of ROS in various cells [26, 38]. To determine whether ROS can stimulate TGFβ1 production in irradiated alveolar epithelial cells, N-acetyl-L-cysteine (5mM, NAC), was incubated for 4 h and then exposed to radiation (8Gy). Pre-incubating cells with NAC blocked radiation-induced ROS generation as described in our previous study [13]. Western blot analysis and densitometric quantification of three independent samples showed that inhibition of radiation-induced ROS production with NAC had effectively reduced the formation of TGFβ1 in the irradiated cells as shown in (Figure 7A and 7B).

**Figure 8:**
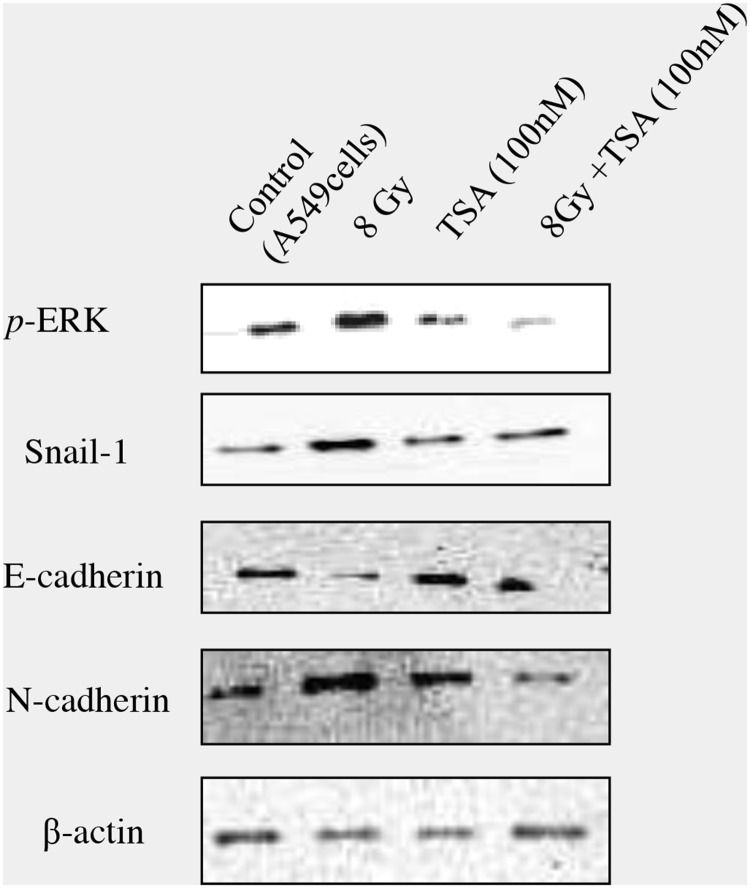
Effect of TSA on irradiated A549 cells In order to confirm the inhibitory effect of TSA on radiation-induced EMT, the effect was tested using A549 cells, a model to study the EMT. TSA (100nM) was incubated 2 hours prior to radiation and western blot analysis on pERK, Snail-1, E-cadherin, N-cadherin was performed and representative blots were shown in the Figure 8.

The data clearly showed that radiation, by enhancing ROS, stimulates TGFβ1 formation which promotes the transition of epithelial cells to undergo mesenchymal characteristics. The effect of TSA was not only tested in irradiated RLE-6TN cells, but also confirmed in another cell line, alveolar epithelial cells (A549). Western blot analysis showed that preincubation of TSA at the dose of 100nM inhibited pERK, Snail-1 and N-cadherin and also improved E-cadherin levels in irradiated A549 cells as shown in Figure 8.

## DISCUSSION

Tissue fibrosis is an important cause for morbidity and mortality worldwide. Fibrosis occurs by increased proliferation of fibroblast and myofibroblast. EMT acts as an intermediatory process during tissue fibrogenesis [39]. Previously, we have reported that radiation can induced EMT in type II alveolar epithelial cells and also in irradiated FVBN mice lungs[12,13]. Therefore, inhibition of EMT might be a rational strategy in intervention of lung fibrosis. Recently, histone deacetylase (HDAC) inhibitors receive lot of researchers’ attention because of its anticancer and antifibrotic potentials in experimental models of cancers and in recent years the applicability of HDAC inhibitors for the treatment of fibrotic disorders has been explored [40].

A HDAC inhibitor, trichostatin A (TSA) has pleiotropic effects targeting key pathological processes including inflammation, proliferation, angiogenesis and fibrosis [41]. In our study, we demonstrate that TSA effectively decreased radiation-induced EMT by blocking ROS/MEK/ERK/GSK3β/Snail signaling pathway and we also elucidate ROS/TGFβ1 signaling mechanism also plays a vital role in radiation induced EMT.

EMT is a process whereby epithelial cells lose its cell to cell contact and gradually loses its epithelial marker and enhance the expression of mesenchymal components [42]. From our morphological study, we observed that radiation stimulated lose of cuboidal nature of type II alveolar epithelial cells and exhibited spindle shaped mesenchymal morphology. On the other hand this transition was effectively blocked by administration of TSA in irradiated alveolar epithelial cells showing inhibitory potential of TSA. E-cadherin, a cell adhesion molecule of epithelial cells, gradually lose its expression during EMT which is an important hall mark of EMT process.

Additionally, α-smooth muscle actin (α-SMA) has been extensively studied during EMT as it is an important myofibroblast marker and shown to increase markedly in epithelial cells that undergo transition [43]. Following the morpholgical observations, we analyzed the expression of both E-cadherin (epithelial marker) and α-SMA in RLE-6TN cells and data suggested that TSA could effectively alter EMT by preserving the expression of E-cadherin and inhibiting α-SMA. TSA was also reported to preserve the membrane localization of E-cadherin and inhibited vimentin, an intermediate filament, during TGFβ1-induced EMT in hepatocytes [44]. In retinal pigment epithelium cells, TSA prevented TGFβ2-induced morphological changes and upregulated the expression of α-SMA, collagen type I & IV, fibronectin (mesenchymal marker) and transcription factor including snail and slug [45].

Snail, a zinc finger transcription factor, function mainly acts as a repressor of E-cadherin expression, thereby stimulate the transition of epithelial cells [46]. In our study, western blot and immunofluorescence assays showed that radiation can effectively stimulate Snail expression and enhance nuclear translocation of Snail protein where it acts as a repressor E-cadherin, while TSA administration down-regulated Snail which in turn inhibits EMT in alveolar epithelial cells. It was shown that treatment with TSA is sufficient to block the repressor effect of Snail in kidney epithelial cells [47]. TSA was reported to downregulate epithelial marker and up regulate mesenchymal marker via targeting Snail-1 and it has been suggested that in human mammary epithelial cells, TSA can effectively inhibit induction and also maintenance of EMT [48].

Glycogen synthase kinase-3 beta (GSK3β), a serine/threonine kinase is active in resting epithelial cells and is necessary to maintain epithelial architecture [49]. In normal cells, GSK3β inhibit snail by phosphorylating it; however, in EMT process GSK3β activity is inhibited which leads to upregulation of Snail and thereby decreases expression of E-cadherin [50]. Previously, we reported that radiation enhance GSK3β phosphorylation at serine 9 which prevent its binding efficiency with Snail and facilitate nuclear translocation of Snail [13]. In this study, we observed that TSA decreased the expression of inactive form of GSK3β which might prevent Snail activation and thereby inhibiting EMT in lung epithelial cells. GSK3β inactivation is stimulated by various upstream signalling pathways including WNT, PI3k/AKT and MAPK [51–53]. Our previous investigation on MAPK to radiation in RLE-6TN cells showed that among other kinases, ERK1/2 signaling involves mainly in deactivating GSK3β via phosphorylation [13]. In this study, the phosphorylation of ERK1/2 in RLE-6TN cells was inhibited by TSA, indicating that TSA administration suppresses ERK/GSK3β/Snail/E-cadherin pathway leading to inhibition of radiation-induced of EMT in irradiated alveolar epithelial cells.

In lung, increased oxidative stress leads to cell damage and triggers inflammation and finally lead to fibrosis [54]. The biological effect of ionizing radiation is mainly caused by formation reactive oxygen species (ROS) which triggers oxidative damage to protein, lipids, DNA and alters signaling pathways [55]. In renal tubular epithelial cells, ROS mediates TGFβ1-induced EMT by mainly activating MAPK [56]. We have shown that ROS activate MEK/ERK signaling in irradiated alveoloar epithelial cells [13].

Our current data provide the first evidence that TSA effectively blocked radiation-induced ROS in lung alveolar epithelial cells and thereby, prevented transition of epithelial cells into mesenchymal phenotype. Yang et al., [57] have reported that TSA can scavenge free radicals and alters Nrf2-ARE signaling in TGFβ- induced myofibroblast differentiation. TSA, by acting an antioxidant reduces EtOH-induced oxidative stress in the human neuronal cell line [58]. As TSA is a specific inhibitor of HDAC activity (Class I and Class II), we next examined whether HDAC proteins are involved in radiation induced alveolar epithelial to mesenchymal transition. Our findings on time dependent response of HDAC proteins showed that radiation did not alter the protein levels of HDAC in RLE-6TN cells, thus we now show for the first time that TSA exerts anti-EMT effect in irradiated RLE-6TN cells mainly by inhibiting radiation-induced ROS production and preventing the propagation of MEK/ERK signaling.

TGFβ is regarded as a key mediator of fibrosis. Epithelial cells derived from a variety of tissues including lung, kidney and breast [59–61] display myofibroblast features following exposure to TGFβ. TGFβ, a central mediator of fibrosis, controls EMT by a network of signaling and transcriptional events [9, 10]. TGFβ is known to directly induce EMT in pulmonary adenocarcinoma cell and also in primary type II epithelial cells [34]. TGF-β1 has been described as the main CTGF inducer [62]. CTGF is a secreted matricellular protein that has been implicated as a regulator of cellular proliferation, angiogenesis and remodeling of the extracellular matrix [63]. CTGF has recently received much attention as a novel profibrotic factor and plays important role in fibrosis and in various diseases including radiation enteritis [64]. *In vitro* studies in lung epithelial cells showed an increased expression of EMT-associated proteins due to blocking of exogenous TGFβ by knocking-down of CTGF, suggesting that CTGF is a mediator in TGFβ-induced EMT [65]. The main finding of our study showed that radiation enhanced the protein levels in both TGFβ and CTGF gradually with increase in the time; whereas TSA inhibited the expression of both proteins in alveolar epithelial cells. Yoshikawa et al., [66] have reported that TSA prevented TGF-β1–induced EMT in human renal proximal tubular epithelial cells. In our study, inhibition of TGFβ using SB431542 inhibited the protein levels of GSK3 β, snail and α-SMA, implying that TGFβ is required for radiation-induced EMT.

ROS generated by ionising radiation has been shown to play a crucial role in development of fibrosis. One of the possible mechanisms whereby ROS induce fibrosis is targeting TGFβ. Increased ROS formation would convert latent form of TGF-β to active form and also stimulate the expression and secretion of TGFβ in many types of cells [37, 67]. In another study, ROS generated by xanthine/xanthine oxidase system was shown to increase TGFβ expression in human alveolar epithelial cells [68]. In our study, suppression of ROS production with antioxidant (NAC) lead to inhibition of TGFβ in irradiated alveolar epithelial cells, suggesting that ROS is necessary to stimulate TGFβ which mediates transition of epithelial cells.

**Figure 9:**
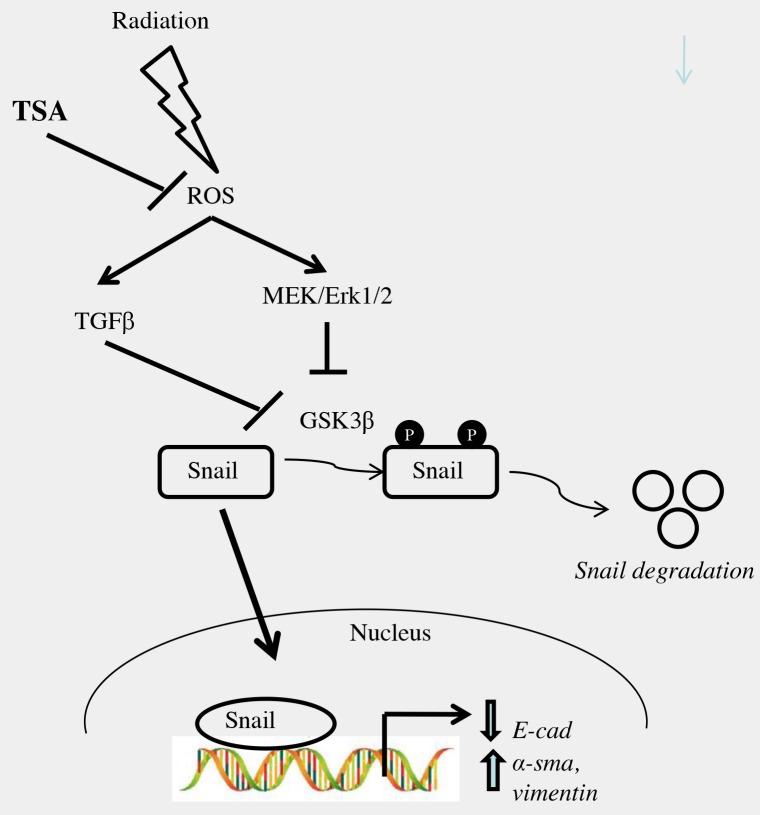
Schematic representation of the proposed mechanism for effect of TSA on radiation-induced EMT in alveolar type II epithelial cells TSA, at least in part, might prevent radiation-induced EMT by blocking i) ROS/MEK/ERK ii) ROS/TGF-β1 signaling pathways in RLE-6TN cells.

In summary, our results showed that TSA inhibits EMT in irradiated alveolar epithelial cells (Figure 9). The inhibitory mechanism of TSA in irradiated alveolar epithelial cells is due to inactivation of ROS/MEK/ERK signaling. In addition, the results showed that radiation exposure leads to increased generation of ROS, which would active TGFβ. Meanwhile, TSA administration attenuated ROS/TGFβ signalling in radiation-induced alveolar EMT. However, the role of TGFβ/smad signaling and CTGF are still unclear in irradiated alveolar epithelial cells. Additional studies on TGFβ/smad and CTGF are needed to fully understand their effects on radiation induced EMT and also upon TSA treatment, which help to understand the therapeutic efficacy of TSA on lung fibrosis.

## MATERIALS AND METHODS

### Cell culture

RLE-6TN cells, a rat alveolar type II epithelial cell line, was obtained from ATCC (Manassas, VA) and routinely maintained in Dulbecco's Modified Eagle Medium (DMEM) containing 10 % fetal bovine serum, 2 mM L-glutamine, 100 IU/mL penicillin and 100 μg/mL streptomycin (purchased from Invitrogen, Carlsbad, CA) at 37° C with 5% CO2 in air.

### Irradiation and TSA treatment

Once cells reached >80% confluence, the DMEM medium was replaced with serum-free medium for 24 h prior to irradiation. Cells were then irradiated with a single dose of 8 Gy γ rays using a 137Cs irradiator at a dose rate of 3.64 Gy/min. All irradiations were performed at room temperature. To analyse the effect of TSA (Sigma-Aldrich, St. Louis, MO), TSA at a final concentration of 100 nM was added to the culture and incubated for two hours prior to radiation at the dose of 8 Gy.

### Western blot analysis

RLE-6TN cells were lysed using RIPA buffer containing 1 m*M* PMSF, 1μg/mL aprotinin, 1μg/mL leupeptin, 1 m*M* Na_3_VO_4_ and 1 m*M* NaF, and stored in aliquots at -80°C until further analysis. The lysate (20 μg) was mixed with an equal volume of sample buffer, denatured by boiling, and then separated on 10-15% polyacrylamide mini-gel. The proteins were transferred to nitrocellulose membranes (Amersham, Arlington Heights, IL), blocked with 5% milk and incubated overnight with E-cadherin (Abcam, Cambridge, MA), Snail (Abcam), *p-*GSK3β (s9) (Cell Signaling Technology, Danvers, MA), GSK3β (Cell Signaling Technology), α-SMA (Abcam), phosphorylated ERK (Santa Cruz Biotechnology), ERK (Santa Cruz Biotechnology), *p*-p38, *p*-JNK (Cell Signaling Technology) and β-actin antibodies (Sigma, St. Louis, MO). The blots were then incubated with anti-mouse or anti-rabbit IgG horseradish peroxidase conjugated antibodies (GE Healthcare, Piscataway, NJ) for 1 h at room temperature. Finally, the signal was detected using Amersham ECL plus (GE Healthcare).

### Reactive oxygen species (ROS) generation

ROS generation was measured using 2′7′-dichlorodihydofluorescein diacetate (H_2_DCFDA, Invitrogen, CA) as described previously [22]. Cells were pre-incubated with TSA for 2 hours followed by 10 μM H_2_DCFDA (in PBS) treatment for 30 min, washed and then irradiated with 8 Gy of ^137^Csγ rays; the cells were then returned to the incubator and maintained for 1 h. The fluorescence intensity was then measured at excitation wavelength 485 nm and emission wavelength 530 nm using a microplate fluorescence reader (Bio-Tek Instruments Inc., Winooski, VT).

### Immunofluorescence staining

Cells grown on 8-well chamber slides were pretreated with TSA followed by 8 Gy radiation of ^137^Cs γ rays and then fixed with 4% neutral formalin for 30 minutes. After washing three times with 1 X PBS, the cells were blocked with 3% BSA for 1h. The cells were then incubated with Snail (Abcam), antibody at 4 ˚C overnight. After washing with PBS, the sections were incubated with Texas Red conjugated anti-rabbit (Vector Laboratories, Burlingame, CA) antibodies at room temperature for 30 min. Nuclei were counterstained with 4’-6-diamidino-2-phenylindole (DAPI), and the slides were analyzed using a fluorescence microscope.

### Immunoprecipitation

Five hundred μg of cell lysate were incubated with 10 μL of the Snail antibody overnight at 4°C in a rotating wheel. After overnight incubation, 50μL of protein A/G agarose were added and the solution incubated for 4 h at 4°C with rotation. The complexes were harvested by centrifugation, washed three times with RIPA buffer and then dissociated from the beads by addition of 50μL of SDS sample loading buffer and heated for 5 min at 95 °C. The protein levels of HDACs were analyzed using Western blot.

### RT-PCR

Total RNA was isolated from cells using an RNeasy Mini Kit according to the manufacturer's recommendations (Qiagen, Valencia, CA, USA) with the addition of DNase digestion with an RNase-free DNase set (Qiagen). One microgram of total mRNA was used as template for cDNA synthesis with the First-Strand cDNA Synthesis Kit (Invitrogen) according to the recommendations of the manufacturer. The PCR amplifications were carried out in 50 μl reaction solution containing 2 μl of RT product, 5 μl of 10× PCR buffer, 0.15 mM MgCl2, 1 μl of 10 mM dNTP, 15 pmol of sense and antisense primers, and 2.5 U Taq polymerase (Promega). The PCR solution was denatured initially at 94 °C for 2 min, followed by 25–30 cycles through a 1-min denaturing step at 94 °C, a 40-s annealing step at 50–55 °C, and a 40-s elongation step at 72 °C. E-cadherin, α-SMA and GAPDH primers were designed and synthesized by Integrated DNA Technologies (Iowa City, IA, USA).

### Statistical analysis

Statistical analysis was performed using one-sample Student's *t* test to compare the differences between the TSA and irradiated groups. A *p* value of ≤ 0.05 was considered significant.
